# Deacclimation and reacclimation processes in winter wheat: novel perspectives from time-series transcriptome analysis

**DOI:** 10.3389/fpls.2024.1395830

**Published:** 2024-05-14

**Authors:** Gabija Vaitkevičiūtė, Andrius Aleliūnas, Gintaras Brazauskas, Rita Armonienė

**Affiliations:** Institute of Agriculture, Lithuanian Research Centre for Agriculture and Forestry, Akademija, Lithuania

**Keywords:** abiotic stress, climate change, cold acclimation, cold hardening, RNA-seq, *Triticum aestivum* L.

## Abstract

Winter wheat achieves freezing tolerance (FT) through cold acclimation (CA) – a process which is induced by low positive temperatures in autumn. The increasing occurrences of temperature fluctuations in winter lead to deacclimation (DEA), causing premature loss of FT, and the cultivars capable of reacclimation (REA) are more likely to survive the subsequent cold spells. The genetic mechanisms of DEA and REA remain poorly understood, necessitating further research to bolster climate resilience in winter wheat. Here, we selected two winter wheat genotypes with contrasting levels of FT and conducted a ten-week-long experiment imitating low-temperature fluctuations after CA under controlled conditions. Crown and leaf tissue samples for RNA-sequencing were collected at CA, DEA, and REA time-points. It is the first transcriptomic study covering both short- and long-term responses to DEA and REA in winter wheat. The study provides novel knowledge regarding CA, DEA, and REA and discusses the gene expression patterns conferring FT under temperature fluctuations. The freezing-tolerant genotype “Lakaja DS” showed elevated photosynthetic activity in leaf tissue and upregulated cryoprotective protein-encoding genes in crowns after CA when compared to the freezing-susceptible “KWS Ferrum”. “Lakaja DS” also expressed cold acclimation-associated transcripts at a significantly higher level after 1 week of DEA. Following REA, “Lakaja DS” continued to upregulate dehydrin-related genes in crowns and exhibited significantly higher expression of chitinase transcripts in leaves, when compared to “KWS Ferrum”. The findings of this study shed light on the genetic mechanisms governing DEA and REA in winter wheat, thus addressing the gaps in knowledge regarding FT under low-temperature fluctuations. The identified genes should be further examined as potential molecular markers for breeding strategies focused on developing freezing-tolerant winter-type crops. Publicly available datasets generated in this study are valuable resources for further research into DEA and REA, contributing towards the enhancement of winter wheat under global climate change.

## 1 Introduction

Winter wheat (*Triticum aestivum* L.) is favored in the temperate zone as a staple crop due to its high productivity and nutritional value (Entz and Fowler, 1991; Wieser et al., 2020). Due to the prolonged vegetation period throughout autumn and winter, the productivity of winter wheat is over 30% higher compared to spring wheat (Entz and Fowler, 1991). The warming climate and milder winters are predicted to provide beneficial conditions for winter wheat (Li et al., 2020; Minoli et al., 2022). However, climate change will shift the cultivation area of winter wheat to higher latitudes, where freezing tolerance (FT) will remain a strong limiting factor for yield. Furthermore, the increasing instances of temperature fluctuations in winter will negatively impact the survival of winter wheat (Rapacz et al., 2014; Vaitkevičiūtė et al., 2022a). To combat this, new insights are required into the factors affecting the acquisition and loss of FT. Lengthy RNA-sequencing (RNA-seq) and metabolomic studies, encompassing multiple tissues, genotypes, and continuous time-points are necessary to provide the broader picture of these processes in winter wheat.

FT is achieved through cold acclimation (CA), also referred to as cold hardening, which occurs at low positive temperatures. CA induces multiple molecular and physiological changes, such as the accumulation of cryoprotective carbohydrates and proteins, delayed development of the shoot apex, and cessation of growth (Trischuk et al., 2014; Li et al., 2018; Zhao et al., 2019). During this period, which can last up to 8 weeks in winter wheat, the plants remain in a vegetative phase (Fowler et al., 1996). Throughout CA, gene expression is affected by transcription factor (TF) proteins, which bind to specific DNA sequences and subsequently either activate or repress the transcription of genes. A group of TFs activated by an endogenous signal can comprise a complex transcriptional cascade. Currently, the inducer of CBF expression (ICE) – C-repeat-binding factor (CBF) – cold-responsive (COR) pathway remains one of the most-studied cold-induced TF pathways in plants (Liu et al., 2019). Cold stress promotes the accumulation of ICE TFs, which activate the transcription of *CBF* genes. The CBF TFs, in turn, upregulate the COR group of proteins, which carry out various protective functions – for example, COR14b protects the photosynthetic apparatus after freezing damage (Rapacz et al., 2008), while COR dehydrins play a cryoprotective role during frost-induced desiccation (Kosová et al., 2011). Therefore, numerous transcriptomic and bioinformatic studies have reported increased expression of the ICE-CBF-COR pathway genes in wheat under cold stress (Li et al., 2018; Guo et al., 2019; Aleliūnas et al., 2020; Pan et al., 2022).

The low positive temperatures, required for CA, also induce a different process in winter-type crops, known as vernalization. Fulfillment of the vernalization requirement allows plants to transition from the vegetative to the reproductive stage (Mahfoozi et al., 2001a). The relationship between CA and vernalization is complex and interlinked, for example, the vernalization locus *Vrn-A1* had been shown to regulate the duration of cold-responsive gene expression (Laudencia-Chingcuanco et al., 2011). Winter wheat reaches its FT peak during mid-winter, however, once vernalization saturation is reached, FT begins to decline (Fowler et al., 1996). Therefore, in the second half of winter, snow cover is extremely important for the overwintering of crops, as snow depth of 8–10 cm provides sufficient insulation for the survival of winter wheat under temperatures as low as -27°C (Armonienė et al., 2013). However, climate change has led to the thinning or even absence of snow cover, thus, leading to winterkill (Vico et al., 2014; Kersebaum, 2022). The CA induction temperature can vary between wheat genotypes, but studies show the threshold to be approximately 10°C (Fowler, 2008). Nevertheless, the increasing temperatures in autumn will delay the induction of CA in the temperate zone, where winter wheat is a major cereal crop. Consequently, the reduced irradiance and the shortened photoperiod, which occur later in the season, will hinder the photosynthetic activity and the subsequent metabolite accumulation of winter wheat, decreasing the efficacy of CA and rendering the crops more susceptible to freezing damage (Rapacz, 1998; Rapacz et al., 2014; Dalmannsdottir et al., 2017; Vaitkevičiūtė et al., 2022a).

While CA is a well-studied process, there is a significant gap in knowledge regarding deacclimation (DEA) and especially reacclimation (REA). DEA naturally occurs in cold-acclimated winter-type crops upon exposure to warmer temperatures. After this transition the physiological and molecular changes which occurred during CA are partly reversed, as the plants lose their FT and resume growth (Mahfoozi et al., 2001b). DEA had been shown to result in decreased antioxidant, soluble carbohydrate, and cryoprotective protein content, as well as increased osmotic potential (Trischuk et al., 2014; Rys et al., 2020; Vaitkevičiūtė et al., 2022b). Moreover, hypoxia response was found to play an important role in early DEA in *Arabidopsis thaliana* (L.) Heynh (Vyse et al., 2022). However, DEA can also occur prematurely in response to warm spells in winter. Resumed growth and elevated water content under increased temperature render the crops more susceptible to freezing injury when exposed to sudden freezing events (Rapacz et al., 2014, 2022; Willick et al., 2021). Due to climate change, the growing prevalence of such temperature fluctuations is especially dangerous when they occur after the plants have reached vernalization fulfillment and started transitioning from the vegetative to the reproductive stage (Laudencia-Chingcuanco et al., 2011). A study by Rapacz et al. (2022) highlights that premature DEA is becoming an increasingly important factor negatively affecting the winter survival of winter wheat and triticale under climate change. However, more research is required to elucidate the molecular processes behind DEA.

Following DEA, FT can be regained through REA, provided that the vernalization requirement had not been fulfilled, and the temperature remains within the low-positive range suitable for REA. Furthermore, the findings of Willick et al. (2021) propose that REA in winter wheat and rye is more feasible after a dry winter thaw, as opposed to a wet winter thaw. Our earlier studies demonstrate that six winter wheat genotypes with different levels of FT are capable of REA, during which they accumulate soluble carbohydrates and antioxidants, and deplete the starch reserves in crown and leaf tissues (Vaitkevičiūtė et al., 2022a, 2022b). The complex network involving vernalization requirement, soluble carbohydrates and cryoprotective proteins may play a significant role in the capacity to reacclimate (Vítámvás and Prášil, 2008; Trischuk et al., 2014), however, the exact mechanisms of REA are still unclear. The ability of winter wheat to reacclimate efficiently will become increasingly crucial for its survival and subsequent productivity in the temperate zone. Ultimately, to combat yield losses under global climate change, new winter crop varieties, tolerant to DEA, and capable of REA, will be in demand.

Our earlier study showed that the FT of cold-acclimated winter wheat decreases significantly after 1 week of DEA at 10°C, and is regained after 2 weeks of REA at 2°C (Vaitkevičiūtė et al., 2022a). The most freezing-tolerant (“Lakaja DS”) and the least freezing-tolerant (“KWS Ferrum”) genotypes from this previous work were selected to conduct a follow-up RNA-seq study under identical controlled CA, DEA, and REA conditions. Thus, we generated an RNA-seq dataset, spanning two winter wheat genotypes, two tissues, six sampling points, and three biological replicates each. This dataset can be utilized to conduct multiple differentially expressed gene (DEG) comparisons and test numerous hypotheses, therefore, it was made publicly available. The aims of this study were (1) to assess the gene expression patterns in winter wheat crown and leaf tissues throughout CA, DEA, and REA, (2) to evaluate the numbers of DEGs during DEA and REA in comparison to CA, and (3) to compare the transcriptomic profiles of genotypes with contrasting FT during CA, DEA, and REA. To the authors’ knowledge, this is the first study encompassing both the crown and leaf tissues of two different winter wheat genotypes throughout CA, DEA, and REA. The findings of this study address the gap in knowledge regarding the processes of DEA and REA.

## 2 Materials and methods

### 2.1 Plant material and growth conditions

Two winter wheat genotypes were chosen for this experiment based on the results of an earlier study: the freezing-tolerant “Lakaja DS” (Lithuania) and the freezing-susceptible “KWS Ferrum” (Germany) (Vaitkevičiūtė et al., 2022a). The LT_30_ (temperature, at which 30% of plants are killed) values of “Lakaja DS” after CA, DEA, and REA were -19.82, -14.91, and -18.33°C, respectively, while the LT_30_ values of “KWS Ferrum” were -14.74, -11.93, and -13.45°C, respectively. The seeds were placed on filter paper in Petri dishes, soaked in water and stored at 4°C for 4 days under dark conditions, then transferred to room temperature for 16 h. The imbibed seeds were subsequently sown into 125 cm^3^ wells of peat moss substrate (Durpeta, Lithuania) in 28-well trays, with 3 seeds per well. The seedlings were grown in a greenhouse at 18°C temperature and 12 h photoperiod until three-leaf stage was reached. The plants were then subjected to CA at 2°C for 7 weeks, DEA at 10°C for 1 week, and REA at 2°C for 2 weeks (**Supplementary Figure 1**) in the phytotron (PlantMaster, CLF Plant Climatics GmbH, Germany). An additional subset of plants was subjected to 8 weeks of CA at 2°C without subsequent DEA or REA. Plants were watered regularly to keep the substrate moist. The phytotron conditions were set to 80% relative air humidity, 200 μmol m^–2^ s^–1^ light intensity and 12-h photoperiod for the entire experiment.

### 2.2 Plant sampling and RNA extraction

Crown and leaf tissue samples were collected in the middle of the photoperiod at the following time-points: 7 weeks of CA (7wCA), 8 weeks of CA, (8wCA), 24 hours of DEA (24hDEA), 1 week of DEA (1wDEA), 24 hours of REA (24hREA), and 2 weeks of REA (2wREA) (**Supplementary Figure 1**). Three biological replicates were collected per genotype and per sampling point, with a single individual plant serving as a replicate for both leaf and crown samples separately. A single replicate of the leaf sample comprised a total of 100 mg leaf cuttings, originating from 2 leaves from the same individual plant. A single replicate of the crown sample weighed 100 mg on average and consisted of a 1 cm^3^ section of the crown region, which is found between the root and the shoot of each individual plant. The samples were placed in DEPC-treated RNase-free Eppendorf tubes and flash-frozen in liquid nitrogen. The samples were ground using the Mixer Mill MM 400 (Retsch, Germany). Total RNA extraction was carried out using the GeneJET Plant RNA Purification Mini Kit according to the producer’s instructions (Thermo Fisher Scientific, Lithuania). To remove genomic DNA, the samples were treated with Dnase I, RNase-free (Thermo Fisher Scientific, Lithuania). Subsequently, RiboLock RNase Inhibitor (Thermo Fisher Scientific, Lithuania) was added at the final concentration of 1 U μl^-1^. RNA quality was initially evaluated using the bleach gel protocol (Aranda et al., 2012), whereas RNA concentration and purity were determined using Nanodrop 1100 (Thermo Scientific, USA).

### 2.3 RNA-seq library preparation, sequencing, and mapping

RNA-seq library preparation and sequencing was carried out at GENEWIZ (Azenta Life Sciences, Germany). The RNA library was prepared using Poly(A) capture with ERCC spike-in and sequenced as paired-end reads (2x150 bp) on Illumina NovaSeq sequencing platform. To fulfill the computational requirements for transcriptome mapping and subsequent gene expression analyses, a server, running CentOS Linux 7, with 2X Intel Xeon Gold 6132 CPU, 768 GB RAM and >10 TB hard drive space was used. The quality of raw reads was evaluated using FastQC v0.11.9 (Andrews, 2023). Sequencing data preprocessing was carried out using fastp v0.23.4 (Chen, 2023): polyG tail trimming was enabled, reads with Phred quality score < 20, length < 25 base pairs (bp), and percentage of unqualified bases < 40 were removed. The reads were mapped using Salmon v1.9.0 (Patro et al., 2017) on spring wheat “Chinese Spring” IWGSC RefSeq v2.1 transcriptome, containing 106 914 high confidence (HC) genes (Zhu et al., 2021).

### 2.4 RNA-seq data analysis

Differential gene expression (DGE) analyses were carried out on R v4.3.1 (R Core Team, 2022) using “DESeq2” v1.40.2 package (Love et al., 2014). The Benjamini-Hochberg Procedure was applied to control the false discovery rate and calculate adjusted p-values (padj). Principal component analyses (PCAs) were likewise carried out using this package.

Gene ontology (GO) biological process (BP), cellular component (CC), molecular function (MF) subontology IDs and descriptions, as well as UniProtKB/TrEMBL IDs were accessed via the R package “biomaRt” v2.56.1 (Durinck et al., 2009), whereas UniProtKB protein descriptions were obtained from the web-based UniProt ID Mapping tool (Huang et al., 2011) (**Supplementary Table 1**). A total of 57 097, 54 957, and 69 093 RefSeq v2.1 gene IDs had corresponding GO BP, CC, and MF IDs, respectively. A total of 101 432 RefSeq v2.1 gene IDs had corresponding UniProt IDs. IWGSC RefSeq v2.1, RefSeq v1.1 and RefSeq v1.0 gene ID correspondences were obtained from the GrainGenes database (Yao et al., 2022).

For subsequent analyses, the data were filtered to only include genes with padj < 0.05 and baseMean > 10, with baseMean representing the average of the normalized count values, divided by size factors, taken over all samples. The package “apeglm” v1.22.1 was used for logarithmic fold change (LFC) shrinkage (Zhu et al., 2019). Heatmaps were generated with the “ComplexHeatmap” v2.16.0 package (Gu, 2022). Digital expression analyses were carried out on Wheat Expression Browser (Borrill et al., 2016), using the available RNA-seq data from wheat abiotic stress studies on drought, heat (Liu et al., 2015), and cold (Li et al., 2015). Volcano plots were drawn via the R package “EnhancedVolcano” v1.18.0 (Blighe et al., 2023). Venn diagrams were drawn using an online tool. (Bioinformatics and Evolutionary Genomics, 2023) GO enrichment was performed and dot plots were generated using “clusterProfiler” v4.8.3 (Wu et al., 2021). The parameters for dot plots were set to show the top 10 suppressed and activated most significantly enriched GO terms each in crown and leaf tissues, however, supplementary tables were generated to contain the additional information which could not be accommodated by figures.

## 3 Results

### 3.1 Gene expression across tissues, genotypes, and sampling points

**Figure 1:**
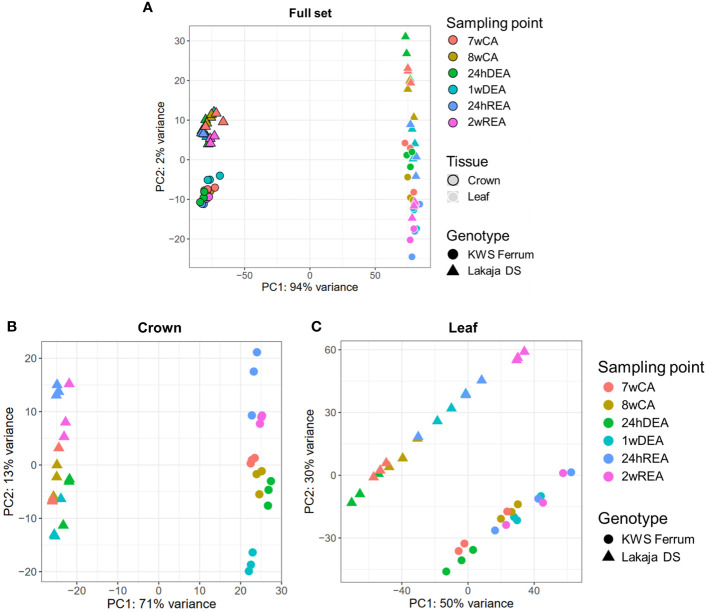
Principal component analyses (PCAs) of gene expression data. Separation of the full set of 72 RNA-seq samples according to sampling point, tissue, and genotype **(A)**. Separation of 36 RNA-seq crown tissue samples according to sampling point and genotype **(B)**. Separation of 36 RNA-seq leaf tissue samples according to sampling point and genotype **(C)**. Three biological replicates were used for every sampling point, tissue, and genotype. 7wCA, 7 weeks of cold acclimation at 2°C; 8wCA, 8 weeks of cold acclimation at 2°C; 24hDEA, 24 hours of deacclimation at 10°C; 1wDEA, 1 week of deacclimation at 10°C; 24hREA, 24 hours of reacclimation at 2°C; 2wREA, 2 weeks of reacclimation at 2°C.

Principal component analyses (PCAs) were carried out to reduce the dimensions and visualize the gene expression data of 106 914 genes in a total of 72 samples across sampling points, tissues, and genotypes (**Figure 1**). Principal component analysis of the entire dataset showed a strong separation between the crown and leaf tissues, with principal component 1 (PC1) accounting for 94% of the variance (**Figure 1A**). PC2 explained 2% of the variance and differentiated the genotypes and sampling points. Two more PCAs were performed to assess the separation of sampling points and genotypes in crown and leaf tissues separately. PC1 and PC2 of the 36 crown tissue samples accounted for 71% and 13% of variation, respectively (**Figure 1B**). PC1 and PC2 of the 36 leaf tissue samples explained 50% and 30% of variation, respectively (**Figure 1C**). In both cases, the strongest distinction was seen between the two genotypes across PC1, whereas the sampling points were separated across PC2. The normalized counts of the genes across all samples can be found in **Supplementary Data Sheet 1**.

### 3.2 Gene expression patterns during cold acclimation, deacclimation, and reacclimation

**Figure 2:**
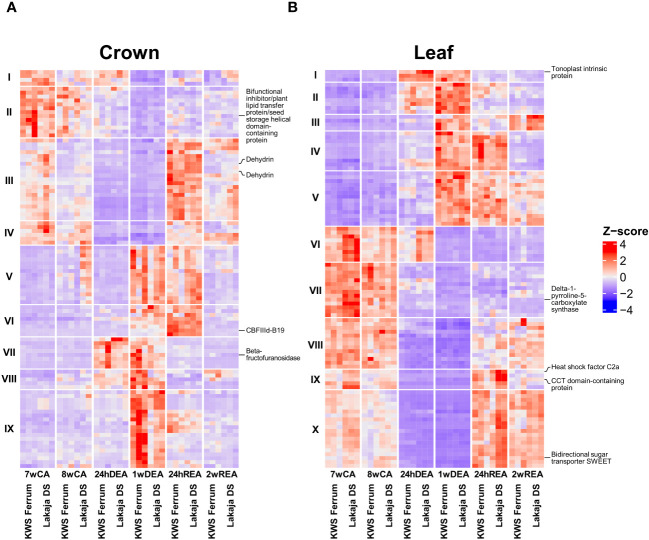
Gene expression throughout cold acclimation (CA), deacclimation (DEA) and reacclimation (REA) in crown **(A)** and leaf **(B)** tissues of two winter wheat genotypes. Depicted are the top 100 most significant genes with the lowest adjusted p-values (padj) and Log2fold change ± 2. Three biological replicates were used for every sampling point, tissue, and genotype. The genes, expressed in crown and leaf tissues were separated according to their expression patterns into IX and X hierarchical clusters, respectively. Protein and GO descriptions corresponding to these genes can be found in **Supplementary Tables 2, 3**. UniProt KB descriptions of five genes were marked in crown **(A)** and leaf **(B)** tissues each; these genes were selected to conduct digital expression analyses (**Supplementary Figure 2**). 7wCA, 7 weeks of cold acclimation at 2°C; 8wCA, 8 weeks of cold acclimation at 2°C; 24hDEA, 24 hours of deacclimation at 10°C; 1wDEA, 1 week of deacclimation at 10°C; 24hREA, 24 hours of reacclimation at 2°C; 2wREA, 2 weeks of reacclimation at 2°C; Z-score, transformed and scaled normalized counts.

Heatmaps were generated to assess the patterns of gene expression throughout CA, DEA, and REA. A filtering step was applied to select the top 100 genes with lowest padj values, Log2fold change ≥ 2 for significantly upregulated and Log2fold change ≤ -2 for significantly downregulated genes in crown and leaf tissue samples throughout CA, DEA, and REA (**Figure 2**). The expression patterns in crown tissue were similar at both 7wCA and 8wCA (**Figure 2A**). At these sampling points, the upregulated genes were grouped into clusters I-IV. The GO BP descriptions of the genes in these clusters include lipid transport, meristem maintenance, protein dephosphorylation, sterol biosynthetic process, as well as responses to abscisic acid, water deprivation, cold acclimation, and water (**Supplementary Table 2**). After 24 hours of DEA, genes, linked to lipid and glycerol metabolic processes, protein folding, and carbohydrate metabolic process were upregulated in cluster VII. Notably, 1 week of DEA induced even stronger changes in gene expression, and genes in clusters V, VII-IX were upregulated. These genes coded for proteins, related to carbohydrate metabolic process, regulation of root development, glutathione metabolic process, protein folding, lignin biosynthetic and catabolic processes, and response to light stimulus. After 24 hours of REA, the genes in cluster V remained upregulated, however, increased expression of genes was likewise observed in the clusters III-IV, VI: their functions included response to abscisic acid, water deprivation, cold acclimation, and water, as well as sterol biosynthetic process, meristem maintenance, protein dephosphorylation, protein folding, and proteolysis. Following 2 weeks of REA, gene expression was less prominent, and clusters III-IV contained upregulated genes, encoding protein dephosphorylation and meristem maintenance proteins. The patterns of gene expression were more prominent in leaf tissue in comparison to crown tissue (**Figure 2B**). Similar patterns were maintained throughout both 7wCA and 8wCA, with the highest expression observed in clusters VI-X. The protein products of these genes play roles in transmembrane transport, proline biosynthetic process, protein phosphorylation and dephosphorylation, meristem maintenance, and regulation of flower development (**Supplementary Table 3**). Twenty-four hours of DEA resulted in increased expression of genes in clusters I-II. Short-term DEA conditions induced elevated levels of transcripts for water transport, spermine and polyamine catabolic processes, as well as protein phosphorylation. 1 week of DEA led to comparatively higher expression of genes, grouped to clusters I-V. The protein products of these genes are involved in transmembrane transport of adenine and guanine, regulation of DNA-templated transcription, protein phosphorylation, response to oxidative stress, and proteolysis. After 24 hours of REA, the expression of genes in clusters IV-V persisted, and the expression of genes in clusters IX-X had increased. These genes code for regulation of flower development, response to heat, hydrogen peroxide, and salt stress, fatty acid metabolic process, and carbohydrate transport. Two weeks of REA led to comparatively diminished expression of genes in clusters V, VIII and X, however, the genes in cluster III were upregulated once again.

Five genes prominently expressed in crown and leaf tissues each were chosen to validate our results via digital expression analysis on Wheat Expression Browser (Borrill et al., 2016) (**Supplementary Figure 2**). The protein products of these genes are likewise marked in **Figure 2**. Two abiotic stress studies, involving drought, heat, and cold were selected to compare our results (Li et al., 2015; Liu et al., 2015). It was found that the same genes were strongly expressed in wheat leaf tissues under abiotic stress. However, a gene coding for a tonoplast intrinsic protein was suppressed after 2 weeks of cold stress, in comparison to the control. Our results likewise show that the transcript for tonoplast intrinsic protein was the most prominent in leaf tissue after 24hDEA and 2wDEA, whereas its expression was reduced after CA and REA (**Figure 2B**).

### 3.3 Changes in gene expression during deacclimation and reacclimation in comparison with cold acclimation

**Figure 3:**
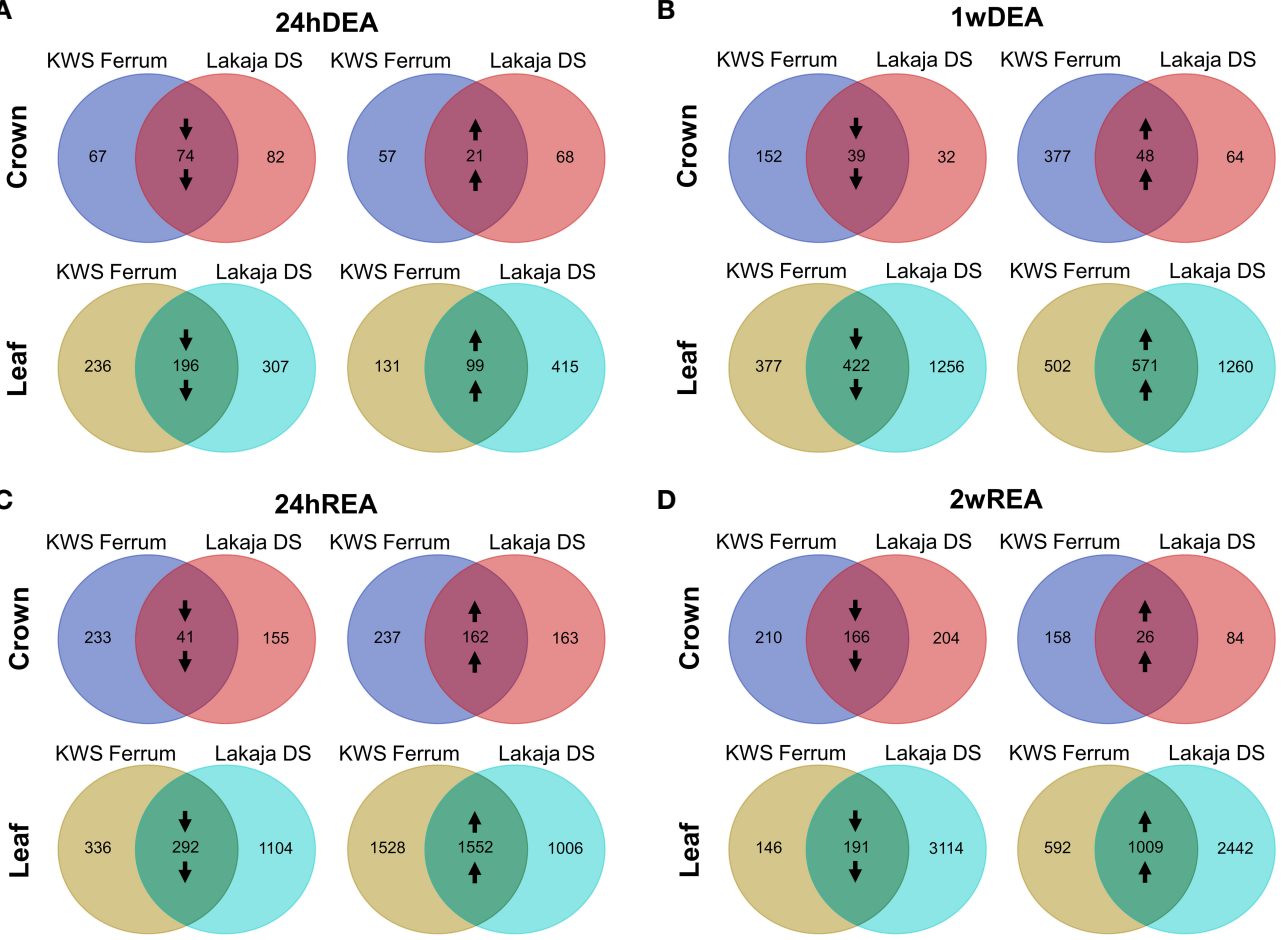
Venn diagrams of differentially expressed genes (DEGs) in crown and leaf tissues of two winter wheat genotypes throughout 24 hours of deacclimation (24hDEA) **(A)**, 1 week of deacclimation (1wDEA) **(B)**, 24 hours of reacclimation (24hREA) **(C)**, and 2 weeks of reacclimation (2wREA) **(D)**. Genes with padj < 0.05, Log2fold change ± 2 are depicted. Downward arrows indicate the downregulation of genes, whereas upward arrows signify the upregulation of genes. All the sampling points were compared to the 7 weeks of cold acclimation (7wCA) sampling point in the corresponding tissue.

To visualize the numbers of DEGs during DEA and REA, the data were compared to the gene expression data of 7wCA. LFC shrinkage was applied for the comparisons, and Volcano plots were generated to visualize these differences (**Supplementary Figure 3**). To incorporate the effect of genotype on gene expression during DEA and REA, Venn diagrams were created (**Figure 3**; **Supplementary Data Sheet 2**). Overall, the numbers of DEGs were consistently higher in leaves than in crowns throughout the experiment. After the initial 24 hours of DEA, more genes were suppressed than activated in both tissues (**Figure 3A**). The opposite occurred after 1 week of DEA, when in both crowns and leaves the number of upregulated genes was higher than downregulated genes (**Figure 3B**). This tendency remained after 24 hours of REA (**Figure 3C**). Notably, after 2 weeks of REA, the crowns exhibited a stronger suppression of gene expression, whereas more genes were activated in leaf tissues (**Figure 3D**).

The freezing-tolerant “Lakaja DS” and the freezing-susceptible “KWS Ferrum” showed different patterns of gene expression throughout DEA and REA. After 24 hours of DEA, “Lakaja DS” had a higher number of differentially expressed genes in crown tissue in comparison to “KWS Ferrum” (**Figure 3A**). However, after 1 week of DEA, an opposite trend occurred, as “KWS Ferrum” had a higher number of both activated and suppressed genes in crown tissue (**Figure 3B**). “Lakaja DS” continued to express a higher number of genes in leaf tissue after both 24 hours and 1 week of DEA. Following 24 hours of REA, “KWS Ferrum” expressed a higher number of genes in the crown in comparison to “Lakaja DS” (**Figure 3C**). This trend remained after 2 weeks of REA. A different pattern was observed in leaf tissue: after 24 hours of REA, “KWS Ferrum” upregulated more genes, whereas “Lakaja DS” suppressed more genes in the leaf tissue. Following 2 weeks of REA, “Lakaja DS” exhibited a higher number of both downregulated and upregulated genes (**Figure 3D**). Detailed information regarding the significantly downregulated and upregulated genes in crown and leaf tissues can be found in **Supplementary Data Sheet 3** and **Supplementary Data Sheet 4**, respectively.

### 3.4 The comparison of expressed genes between genotypes with contrasting freezing tolerance

To compare the expression of genes between the freezing-tolerant genotype “Lakaja DS” and the freezing-susceptible “KWS Ferrum”, GO enrichment analyses were carried out using the descriptions of BP subontology. After 7 and 8 weeks of CA, crown tissue of “Lakaja DS” had activated the transcription of genes, associated with cold acclimation, response to abscisic acid, and response to water, and suppressed the expression of genes, linked to phenylpropanoid biosynthetic process and response to stimulus (**Supplementary Figure 4**; **Supplementary Data Sheet 5**). The “response to stimulus” BP subontology contained multiple genes, coding for WRKY domain-containing proteins and WRKY TFs. Concurrently, the leaf tissue of “Lakaja DS” had activated groups of genes, acting in photorespiration, light harvesting, response to light stimulus, reductive pentose-phosphate cycle, and carbon fixation, and suppressed the genes, coding for the processes of light reaction, regulation of salicylic acid biosynthetic process, cell surface receptor signaling pathway, and response to stimulus.

**Figure 4:**
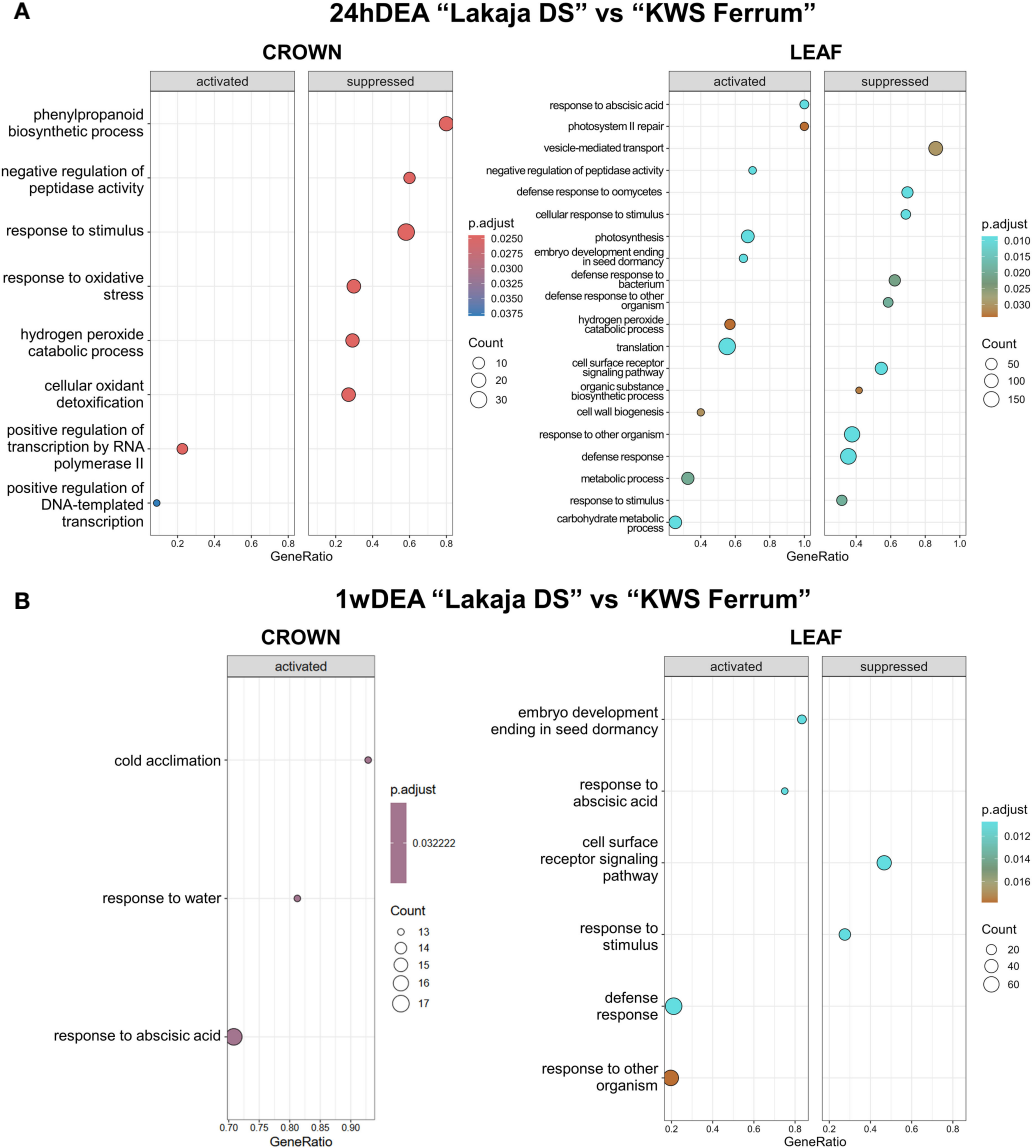
Gene ontology (GO) analysis of gene expression data in crown and leaf tissue of “Lakaja DS” compared to “KWS Ferrum” after 24 hours of deacclimation (24hDEA) **(A)** and 1 week of deacclimation (1wDEA) **(B)**. BP (biological process) terms were used. p.adjust, adjusted p-value; count, the number of genes in the expressed gene-set; GeneRatio, the ratio of number of genes in the expressed gene-set to the number of all genes in the full gene-set.

Twenty-four hours of DEA in “Lakaja DS” crowns resulted in significantly higher enrichment of gene sets, involved in transcription by RNA polymerase II and DNA-templated transcription, whereas phenylpropanoid biosynthetic process, response to stimulus, response to oxidative stress, hydrogen peroxide catabolic process, and cellular oxidant detoxification processes were suppressed, in comparison to “KWS Ferrum” (**Figure 4A**). Leaves exhibited increased expression of gene sets of response to abscisic acid, photosynthesis, embryo development ending in seed dormancy, hydrogen peroxide catabolic process, translation, cell wall biogenesis, and carbohydrate metabolic process groups, and the suppression of cellular response to stimulus, cell surface receptor signaling pathway, and organic substance biosynthetic process groups. In comparison to 24hDEA, 1 week of DEA yielded a smaller number of enriched gene sets between the two genotypes (**Figure 4B**). The crown of “Lakaja DS” activated the genes, related to cold acclimation, response to water, and response to abscisic acid; no gene sets were significantly suppressed. Moreover, the leaf tissue of “Lakaja DS” upregulated the transcripts, linked to embryo development ending in seed dormancy, and response to abscisic acid, while cell surface receptor signaling pathway and response to stimulus processes were suppressed.

**Figure 5:**
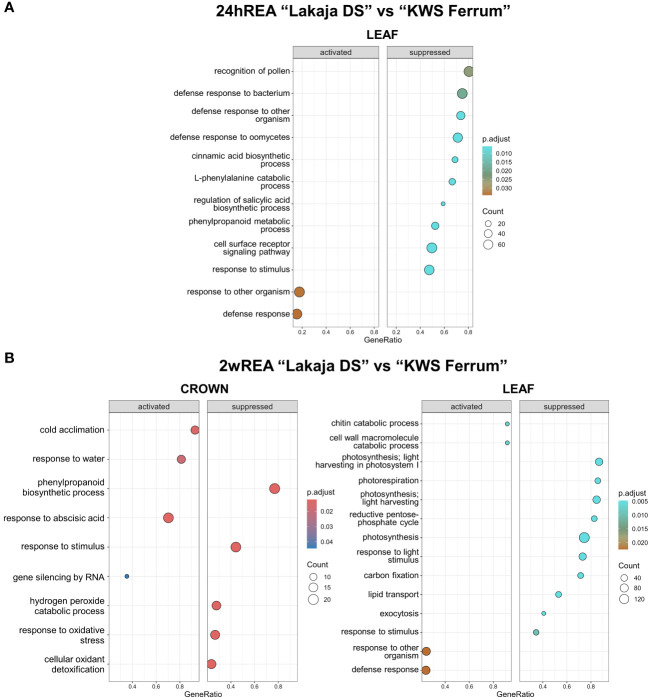
Gene ontology (GO) analysis of gene expression data in crown and leaf tissue of “Lakaja DS” compared to “KWS Ferrum” after 24 hours of reacclimation (24hREA) **(A)** and 2 weeks of reacclimation (2wREA) **(B)**. BP (biological process) terms were used. p.adjust, adjusted p-value; count, the number of genes in the expressed gene-set; GeneRatio, the ratio of number of genes in the expressed gene-set to the number of all genes in the full gene-set.

Strikingly, after 24 hours of REA, no significant changes were observed in the crown tissue of “Lakaja DS” in comparison to “KWS Ferrum” (**Figure 5A**). The leaf tissue of “Lakaja DS” exhibited a significant suppression of genes, associated with the following GO terms: recognition of pollen, cinnamic acid biosynthetic process, L-phenylalanine catabolic process, regulation of salicylic acid biosynthetic process, phenylpropanoid metabolic process, cell surface receptor signaling pathway, and response to stimulus. After 2 weeks of REA, the crown tissue of “Lakaja DS” showed an upregulation of genes, linked to cold acclimation, response to water, response to abscisic acid, and gene silencing by RNA, whereas genes, acting in phenylpropanoid biosynthetic process, response to stimulus, hydrogen peroxide catabolic process, response to oxidative stress, and cellular oxidant detoxification were suppressed (**Figure 5B**). In comparison to “KWS Ferrum”, the leaves of “Lakaja DS” activated genes, involved in chitin catabolic process, cell wall macromolecule catabolic process, and suppressed genes, playing roles in photosynthesis, photorespiration, light harvesting, reductive pentose-phosphate cycle, response to stimulus, response to light stimulus, carbon fixation, lipid transport, and exocytosis. There was a large overlap between the genes, sorted into “chitin catabolic process” and “cell wall macromolecule catabolic process” groups, with most of them encoding a chitinase (EC 3.2.1.14) (**Supplementary Data Sheet 5**).

## 4 Discussion

The increasing occurrences of temperature fluctuations during winter result in premature DEA in winter wheat, consequently reducing their FT capacity (Rapacz et al., 2014, 2022). The inability to rapidly reacclimate prior to sudden drops in temperature results in freezing damage and winterkill. The combination of freeze-thaw cycles and the thinning or even absence of snow cover was shown to negatively impact crop yields (Vico et al., 2014; Kersebaum, 2022). Therefore, to avoid crop losses under global climate change, varieties with improved tolerance to DEA and the ability to swiftly undergo REA will be in demand. Despite this, the number of studies regarding the molecular mechanisms of DEA and REA in winter-type crops are still limited, and to the authors’ knowledge, there are currently no RNA-seq studies on DEA and REA in winter wheat. Due to the labor- and cost- intensive nature of RNA-seq experiments, the studies on DEA and REA are often fragmented, involving only a single tissue type, single genotype, or limited to either DEA or REA (Pagter et al., 2017; Aleliūnas et al., 2020; Horvath et al., 2020). Here, we conducted a lengthy experiment with two winter wheat genotypes, encompassing CA, short-term and long-term DEA, as well as short-term and long-term REA conditions. In the interests of obtaining a fuller perspective on gene expression dynamics throughout these stages, a choice was made to include both crown and leaf tissue samples. Combined, these samples represent both the sink and source tissues, respectively. The interaction between these tissues is an important element in abiotic stress response, as source tissues produce sugars through photosynthesis, and sink tissues either store these sugars or use them as substrates for the biosynthesis of protective metabolites (Dong and Beckles, 2019). Moreover, the crown tissue contains actively dividing shoot apical meristem cells – the survival of these cells is paramount to the regrowth of the plant after winter (Tanino and McKersie, 1985).

Our earlier study showed a strong separation between the metabolite profiles of winter wheat crown and leaf tissues throughout CA, DEA, and REA (Vaitkevičiūtė et al., 2022a). While metabolite profiling studies are informative, they are limited by the availability of metabolite-specific assays and the time it takes to conduct them. RNA-seq, however, represents the plethora of genes, expressed at the time of sample collection. Here, we likewise observed a strong separation of gene expression data between crown and leaf tissues (**Figure 1**). Genotype was the second strongest factor of data separation. While both “KWS Ferrum” and “Lakaja DS” are winter-type wheat cultivars, their FT levels were shown to be significantly different after 7 weeks and 8 weeks of CA at 2°C, 1 week of DEA at 10°C, and 2 weeks of REA at 2°C, with “Lakaja DS” consistently being the more freezing-tolerant genotype (Jaškūnė et al., 2022; Vaitkevičiūtė et al., 2022a). Despite the rapid growth of databases and the availability of the updated wheat reference genome (Zhu et al., 2021), many wheat genes remain uncharacterized, lacking UniProt and GO descriptions. Moreover, a considerable number of such annotations are computationally generated and remain unreviewed (Bateman et al., 2023). Thus, RNA-seq studies inevitably yield sets of genes with unknown functions. However, the results of broader exploratory studies provide the much-needed information for subsequent research into the precise function of single genes. In this study, we present a large collection of DEGs across CA, DEA, and REA (**Supplementary Tables 2,3**; **Supplementary Data Sheets 2-5**). While some of these DEGs currently have unknown functions, they can be selected for future studies and examined for possible potential in the breeding of climate-resilient winter-type crops. Henceforth, the patterns of gene expression in winter wheat crown and leaf tissues throughout CA, DEA, and REA will be discussed, and the genotype-specific transcriptomic profiles will be analyzed.

### 4.1 The freezing-tolerant genotype maintains photosynthetic activity after cold acclimation

**Figure 6:**
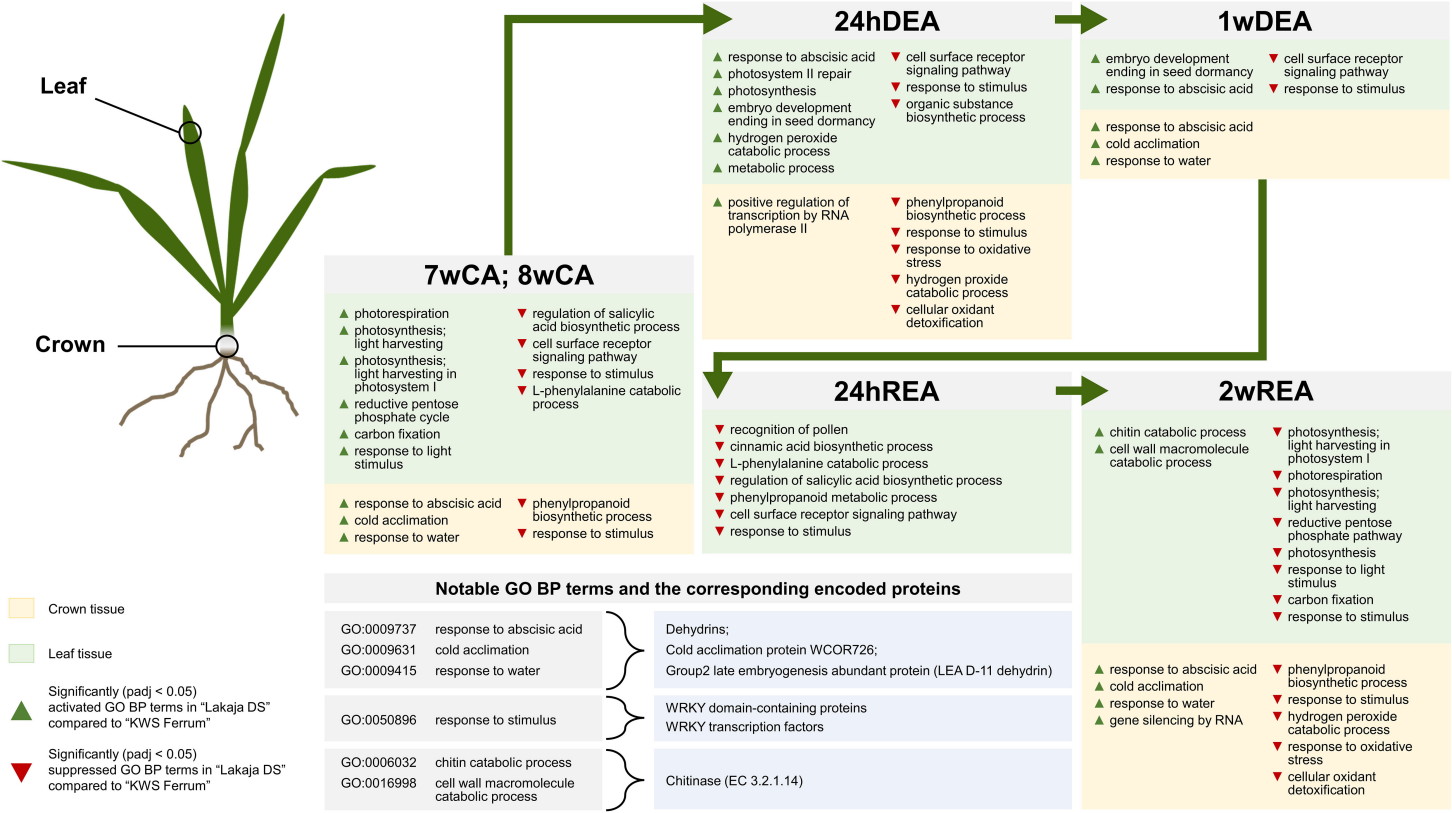
The main results of gene ontology biological process (GO BP) gene enrichment analyses in “Lakaja DS” crown and leaf tissues compared to “KWS Ferrum” throughout 7 weeks of cold acclimation (7wCA), 8 weeks of cold acclimation (8wCA), 24 hours of deacclimation (24hDEA), 1 week of deacclimation (1wDEA), 24 hours of reacclimation (24hREA), and 2 weeks of reacclimation (2wREA). Only statistically significant (padj < 0.05) changes are depicted.

The freezing-tolerant genotype “Lakaja DS” showed increased activities of photorespiration, photosynthesis, reductive pentose phosphate cycle, and carbon fixation after 7 and 8 weeks of CA in comparison to the less freezing-tolerant “KWS Ferrum” (**Figure 6**). Hence, maintained photosynthetic activity under unfavorable conditions could be interpreted as an indicator of improved resilience – freezing-tolerant wheat cultivars had previously been shown to maintain enhanced photosynthetic performance in comparison to freezing-susceptible cultivars (Dahal et al., 2012; Zhang et al., 2015; Hüner et al., 2016). A future study could be conducted to measure the photosynthetic activity in the leaves of winter wheat with different levels of FT throughout CA, DEA, and REA. Persistent photosynthetic activity in leaf tissue under low temperature allows plants to effectively synthesize and accumulate FT-related metabolites. High levels of specific cryoprotective proteins, such as the previously discussed dehydrins, accumulated in wheat crown tissue, strongly correlate with improved FT (Kosová et al., 2013; Vítámvás et al., 2019). Concurrently, in this study “Lakaja DS” showed a significantly higher expression of dehydrins, COR, and RAB proteins. Furthermore, this genotype suppressed the transcripts of the “response to stimulus” BP subontology group (GO:0050896) in both crowns and leaves after CA in comparison to “KWS Ferrum”. This group contained multiple WRKY domain-containing proteins, WRKY45-like TFs, and several WRKY TFs, which had been previously reported to enhance stress tolerance in plants (Qiu and Yu, 2009; Jiang et al., 2017). For example, the overexpression of *CdWRKY2* from *Cynodon dactylon* (L.) Pers. resulted in enhanced FT and upregulated expression of sucrose synthesis-related genes in *A. thaliana* (Huang et al., 2022). Similarly, transgenic *A. thaliana* plants, containing wheat *TaWRKY* genes, exhibited improved tolerance to salt, drought, and cold (Niu et al., 2012). “Lakaja DS” had previously been shown to be more freezing-tolerant than “KWS Ferrum” after CA, DEA, and REA under identical experimental conditions (Vaitkevičiūtė et al., 2022a). However, our results demonstrate that “Lakaja DS” expressed lower levels of *WRKY*-type genes not only after CA, but also after DEA and REA. The enhanced FT of “Lakaja DS” could be determined by upstream genes, which leads to *WRKY* genes becoming less essential, whereas “KWS Ferrum” experiences cold stress more acutely, and therefore increases the expression of *WRKY*-related transcripts to compensate for this drawback. However, the exact order of *WRKY*-type gene expression within the larger network of FT-linked genes is currently unknown and further studies will be required to test this hypothesis.

Our results show that CA induces the upregulation of genes, linked to responses to abscisic acid, cold acclimation, and water in crown and leaf tissues of both genotypes. The genes were activated at significantly higher levels in “Lakaja DS” in comparison to “KWS Ferrum”. Their translated products included the cold acclimation protein WCOR726, multiple dehydrins, a HVA22-like protein, and a Rab protein (**Supplementary Data Sheet 5**). These groups of genes had previously been linked to CA (Laudencia-Chingcuanco et al., 2011; Li et al., 2018; Aleliūnas et al., 2020). The wheat cold-responsive protein WCOR726 belongs to a group of cold-responsive (COR) proteins, which are a part of the ICE-CBF-COR pathway (Liu et al., 2019). An earlier study had likewise shown that WCOR726 is expressed at higher levels in the freezing-tolerant winter wheat “Mironovskaya 808”, in comparison to the freezing-susceptible spring wheat “Chinese Spring” (Kobayashi et al., 2004). Dehydrins (DHN), late embryogenesis-abundant (LEA) and responsive to abscisic acid, ABA (RAB) proteins comprise large families of stress-responsive proteins, involved in salinity, drought, and low temperature tolerance (Tsuda et al., 2000; Chakraborty and Roychoudhury, 2022). Dehydrins are highly hydrophilic proteins from the late embryogenesis abundant (LEA) D-11 family. These proteins were shown to play an important role in the cold acclimation of the Siberian spruce, which is an extremely freezing-tolerant species (Kjellsen et al., 2013). Moreover, the accumulation of dehydrins prior to vernalization fulfillment had been associated with enhanced winter survival in the field in wheat and barley (Vítámvás et al., 2019). Both HVA22 and Rab proteins were likewise shown to be cold-responsive in barley and wheat (Tsuda et al., 2000; Chen et al., 2002).

### 4.2 Short-term and long-term responses to deacclimation conditions yield different transcriptional profiles in wheat

The initial 24 hours of DEA resulted in a strong suppression of transcripts in both crown and leaf tissues. Earlier studies on short-term response to DEA conditions showed the downregulation of multiple cold-induced genes, for example, those, involved in the ICE-CBF-COR pathway (Byun et al., 2014; Pagter et al., 2017; Vyse et al., 2019). Once more, “Lakaja DS” exhibited lower levels of stress at the RNA level in comparison to “KWS Ferrum”, as the enriched gene sets for response to oxidative stress, hydrogen peroxide catabolic process, and cellular oxidant detoxification were suppressed, and gene sets for transcription were upregulated in crown tissue (**Figure 6**). Meanwhile, the leaf tissue of “Lakaja DS” had upregulated genes, associated with response to abscisic acid, photosynthesis, photosystem II repair, carbohydrate metabolic process, and hydrogen peroxide catabolic process. Oxidative stress is caused by the accumulation of reactive oxygen species (ROS), such as hydrogen peroxide (H_2_O_2_), which damage the molecular plant machinery unless they are properly scavenged (Gill and Tuteja, 2010). Photosynthesis is known to generate large amounts of ROS, therefore, plants are naturally adapted to scavenge these molecules via specialized enzymes and antioxidants (Mittler et al., 2004). However, abiotic stress leads to significantly increased ROS accumulation (Anjum et al., 2017; Hasanuzzaman et al., 2018) and thus, stress-tolerant genotypes are usually more effective at ROS scavenging (de Freitas et al., 2019; Jan et al., 2023). In this case, in comparison to “KWS Ferrum”, “Lakaja DS” maintained a higher level of photosynthetic and metabolic activity while combating the accumulation of H_2_O_2_ in leaf tissue. Meanwhile, ROS scavenging activity in the crown tissue was lower in “KWS Ferrum”.

After 1 week of DEA, the opposite trend in gene expression was observed, as more genes were upregulated than suppressed in both crown and leaf tissues. Furthermore, the gene expression patterns were visually distinctive between the short-term and long-term stages of DEA (**Figure 2**). Long-term DEA led to the upregulation of genes, involved in the carbohydrate and glutathione metabolic processes, regulation of root development, and lignin biosynthetic and catabolic processes in crown tissue, whereas the upregulated transcripts in leaf tissue were linked to response to oxidative stress, protein phosphorylation, and DNA transcription. Thus, increased temperature during long-term DEA resulted in the reallocation of resources towards growth. Earlier studies had shown that DEA results in growth resumption in winter oilseed rape and winter wheat (Rapacz, 2002; Vaitkevičiūtė et al., 2022a). Kutsuno et al. (2023) likewise reported the elongation of roots and increased leaf area, accompanied by the metabolization of soluble carbohydrates in *A. thaliana* plants following DEA. In comparison to “KWS Ferrum”, the crown tissue of “Lakaja DS” had upregulated three sets of genes, associated with cold acclimation, response to water, and response to abscisic acid (**Figure 6**) – these genes mostly code for dehydrins (**Supplementary Data Sheet 5**). In response to higher temperature during DEA, the water uptake must be increased to rehydrate the plant cells and to support elevated metabolic activity (Rys et al., 2020). Therefore, the increased transcription of cold acclimation-, response to water-, and response to abscisic acid-related genes may comprise a mechanism to compensate for water uptake problems after the prolonged growth under low temperature conditions. Notably, the leaves of “Lakaja DS” significantly activated the transcripts of genes, involved in embryo development ending in seed dormancy (GO:0009793). These genes code for uncharacterized proteins, and further inspection using available UniProt tools revealed sequence similarities to LEA type 1 family proteins. As discussed previously, the LEA protein family contains stress-responsive proteins, such as dehydrins. Earlier studies have shown a correlation between the transcript and protein accumulation levels of dehydrins, and the FT of winter-type crops (Kosová et al., 2021). Our research indicates that the more freezing-tolerant genotype is able to persistently express genes associated with CA even after 1 week of DEA. This ability to maintain the expression of CA-related genes could contribute to enhanced FT under fluctuating temperatures.

The analysis of top DEGs showed that the gene *TraesCS3D03G1192600*, which encodes a tonoplast intrinsic protein, was strongly upregulated in winter wheat leaf tissue after both 24 hours and 1 week of DEA (**Figure 2B**). Vyse et al. (2019) likewise reported that after 24 hours of DEA, the expression of a gene, coding for a gamma tonoplast intrinsic protein was upregulated, while several *COR* genes were downregulated in *A. thaliana*. The digital expression analysis on Wheat Expression Browser (Borrill et al., 2016) revealed the increased expression of this gene under control conditions, when compared to cold stress (Li et al., 2015) (**Supplementary Figure 2**). Moreover, drought and heat stress also resulted in the upregulation of this transcript (Liu et al., 2015). The GO BP IDs of *TraesCS3D03G1192600* indicate functions in transmembrane and water transport (GO:0055085; GO:0006833). During DEA, the concentrations of soluble cryoprotective compounds in plant cells decreases, and thus water transport activity is increased to rehydrate the tissues, increase the osmotic potential, and maintain normal cellular functioning (Rys et al., 2020).

### 4.3 The freezing-tolerant genotype reaccumulates dehydrins in crown tissue during reacclimation

Two genes were strongly upregulated during REA and therefore were chosen for subsequent digital gene expression analyses. Short-term 24-hour REA increased the expression of the gene *TraesCS5B03G0791100*, encoding a CBFIIId-B19 protein in winter wheat crown tissue (**Figure 2A**). CBFIIId-B19 is a TF, belonging to the CBF family, which is a part of the ICE-CBF-COR pathway (Liu et al., 2019). An earlier study had identified this gene as a potential target for improving FT in wheat (Tchagang et al., 2017), and the transcription of this gene had been shown to increase in winter wheat leaves after 24 hours of low temperature stress at 2°C (Aleliūnas et al., 2020). The digital gene expression analysis likewise showed significant upregulation of the corresponding RefSeq v1.0 transcript of *TraesCS5B03G0791100* under cold stress (**Supplementary Figure 2**) (Li et al., 2015). According to our results, the expression of this gene was the highest after 24hREA, while it was considerably lower after 7wCA, 8wCA, and 2wREA. This information, combined with the results of other studies suggests that CBFIIId-B19 is involved in response to specifically short-term cold stress. Another gene – *TraesCS3A03G0654500*, encoding a bidirectional sugar transporter SWEET, showed increased expression after 7 and 8 weeks of CA, and especially high expression after both 24hREA and 2wREA in leaves. Digital expression analysis likewise showed strong upregulation of *TraesCS3A03G0654500* in cold-treated wheat leaves (Li et al., 2015). The sugar will eventually be exported transporter (SWEET) family proteins facilitate bidirectional movement of carbohydrates through cell plasma membranes. As reviewed by Ji et al. (2022), *SWEET* transcripts are upregulated in response to abiotic stress, such as high salinity, drought, and cold in *A. thaliana*. Soluble sugars act as cryoprotective molecules, however, they are usually produced in the photosynthetic tissues – subsequently, effective transportation of sugars to different tissues is required to protect plant cells from freezing damage (Yao et al., 2020; Livingston et al., 2021). According to Trischuk et al. (2014), recovery of FT in winter oilseed rape and winter wheat is highly dependent on the reaccumulation of soluble carbohydrates. A more recent study affirmed this by reporting the increased accumulation of soluble carbohydrates in winter wheat crown and leaf tissues after REA (Vaitkevičiūtė et al., 2022a).

In this study, 24 hours of REA led to the upregulation of dehydrin genes, involved in response to abscisic acid, cold acclimation, and water deprivation in the crowns. At this stage, no significant differences were found between the enriched GO BP terms in “Lakaja DS” and “KWS Ferrum” crown tissue. However, the leaf tissue of “Lakaja DS” suppressed the genes, coding for cinnamic acid biosynthetic process, regulation of salicylic acid biosynthetic process, L-phenylalanine catabolic process, and phenylpropanoid metabolic process (**Figure 6**). These results suggest that the initial response in leaves, not crowns, may be a determining factor in the efficacy of subsequent REA and eventual FT in winter wheat. The majority of genes in the “regulation of salicylic acid biosynthetic process” (GO:0080142) set encoded calmodulin-binding proteins. Plant-specific calmodulin-binding proteins have been associated with response to abiotic stress, such as cold, drought, and heat (Singh and Virdi, 2013; Iqbal et al., 2022). The gene sets of “cinnamic acid biosynthetic process” (GO:0009800), “L-phenylalanine catabolic process” (GO:0006559), and “phenylpropanoid metabolic process” (GO:0009698) contained transcripts for a phenylalanine ammonia-lyase (PAL) (EC 4.3.1.24). This enzyme catalyzes the deamination of phenylalanine into cinnamic acid – Lv et al. (2022) had reported an increase *PAL* transcripts, as well as the accumulation of cinnamic acid in leaves of cold-treated wheat plants. In this case, it is interesting that “Lakaja DS” suppressed the genes related to CA, in comparison to “KWS Ferrum”. It could be an indication that “Lakaja DS” undergoes lower levels of stress and consequently does not require high concentrations of calmodulin-binding or PAL proteins.

After 2 weeks of REA, the leaves of “Lakaja DS” suppressed genes, related to photosynthesis, photorespiration, and reductive pentose-phosphate cycle, in comparison to “KWS Ferrum” (**Figure 6**). Strikingly, this is the opposite pattern to the one seen at CA, where “Lakaja DS” maintained elevated photosynthetic activity. However, this time it had activated the enriched GO sets of chitin catabolic process (GO:0006032) and cell wall macromolecule catabolic process (GO:0016998), which upon further inspection contained genes, coding for chitinase (EC 3.2.1.14). Earlier studies had described chitinases exhibiting ice-binding activity in winter-type crops and coniferous species; these proteins were accumulated in leaf and needle tissues during CA and were suggested to play a role in FT (Yeh et al., 2000; Jarząbek et al., 2009). To the authors’ knowledge, there are currently no studies regarding the role of chitinases in REA. Our results show that they are potentially involved in the enhancement of FT after REA, therefore, future studies should be carried out to validate the function of the genes, encoding chitinases. Importantly, the crown tissue of “Lakaja DS” once again showed enhanced transcription of genes, associated with cold acclimation, response to water, and response abscisic acid, in comparison to “KWS Ferrum”. The protein products of these genes included dehydrins, LEA D-11, and WCOR726, and are generally associated with CA and improved FT (Kobayashi et al., 2004; Kjellsen et al., 2013; Vítámvás et al., 2019). Furthermore, the enriched GO BP sets of hydrogen peroxide catabolic process, response to oxidative stress, and cellular oxidant detoxification were suppressed, thus indicating a lower state of ROS-induced stress in the crown cells of “Lakaja DS”. These results indicate that the more freezing-tolerant genotype is able to maintain a favorable environment in its crown tissue following REA, which ensures the continued survival of shoot apical meristem cells.

## 5 Conclusions

This study contributes novel insights into transcriptomic changes in winter wheat crown and leaf tissues throughout CA, DEA, and REA. The inclusion of short- and long-term response to DEA and REA sampling points provides a valuable understanding of the differences between the initial and prolonged effects of temperature fluctuations on cold-acclimated winter wheat transcriptome. Significant differences were detected between the transcriptome profiles of the freezing-tolerant genotype “Lakaja DS” and the freezing-susceptible “KWS Ferrum” at all sampling points. The enhanced FT of “Lakaja DS” under low-temperature fluctuations is controlled by a complex gene network, comprising elevated photosynthetic activity in leaf tissue during CA and the ability to control oxidative stress and maintain a high-level expression of cryoprotective genes in crowns throughout DEA and REA. Strong upregulation of chitinase transcripts was observed in “Lakaja DS” leaves during long-term REA compared to “KWS Ferrum”. The role of chitinases in the reacquisition of FT during REA should be taken into consideration for further studies. Notably, “Lakaja DS” consistently suppressed the transcription of WRKY-related genes at all stages of the experiment when compared to “KWS Ferrum”. WRKY-type TFs are known as positive regulators of FT; thus, the results suggest that enhanced FT throughout CA, DEA, and REA is mainly determined by the genetic mechanisms upstream of WRKY genes. Our study provides valuable datasets covering gene expression patterns in winter wheat crown and leaf tissues under low-temperature fluctuations. The genes identified in this study can be investigated for the potential applications in marker-assisted selection or utilized as targets for genome editing. These findings will contribute to the development of climate-resilient winter wheat, adapted to the temperate zone and its changing climate.

## Data availability statement

The datasets generated in this study are included in the article/ **Supplementary Material**. **Supplementary Data Sheet 1**, containing normalized RNA-seq read counts across samples has been deposited at figshare https://doi.org/10.6084/m9.figshare.25335412.v1. Further inquiries can be directed to the corresponding authors.

## Author contributions

GV: Formal analysis, Investigation, Methodology, Software, Visualization, Writing – original draft. AA: Formal analysis, Investigation, Methodology, Software, Writing – review & editing. GB: Funding acquisition, Resources, Writing – review & editing. RA: Conceptualization, Funding acquisition, Methodology, Project administration, Resources, Supervision, Writing – review & editing.
