# Supplementary material for: Deacclimation and reacclimation processes in winter wheat: novel perspectives from time-series transcriptome analysis
Source: Front Plant Sci. 2024 May 14;15:1395830. doi: 10.3389/fpls.2024.1395830 (PMC11130478; doi:10.3389/fpls.2024.1395830)
Supplement: Supplementary file 1 [file DataSheet_1.zip › Supplementary Figures 1-4.pdf]

Supplementary Material

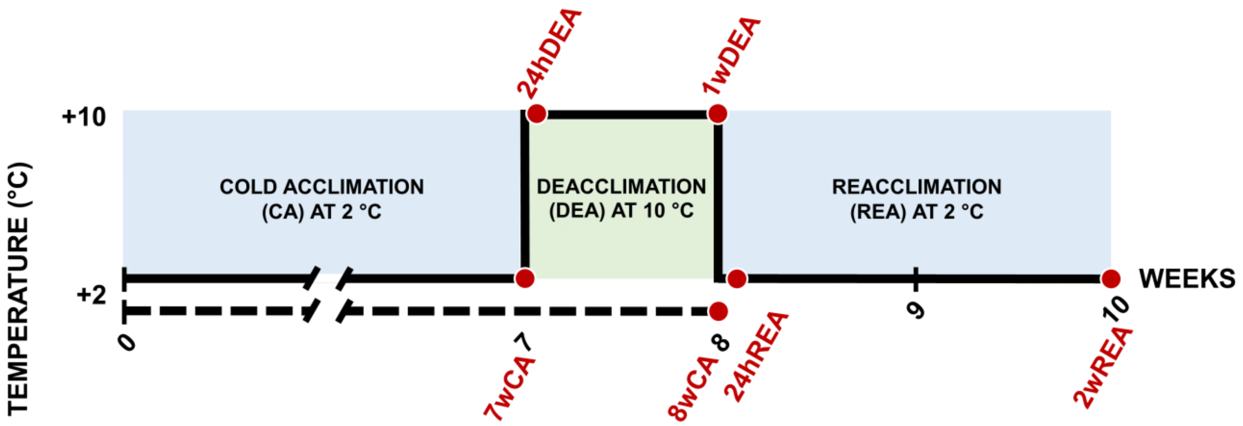

Supplementary Figure 1. The scheme of the cold acclimation (CA), deacclimation (DEA), and reacclimation (REA) experiment. The red dots denote sampling points. The dotted line represents a subgroup of winter wheat, which underwent 8 weeks of CA at 2 °C and no subsequent DEA or REA. 7wCA – 7 weeks of cold acclimation at 2 °C, 8wCA – 8 weeks of cold acclimation at 2 °C; 24hDEA – 24 hours of deacclimation at 10 °C; 1wDEA – 1 week of deacclimation at 10 °C; 24h REA – 24 hours of reacclimation at 2 °C; 2wREA – 2 weeks of reacclimation at 2 °C.

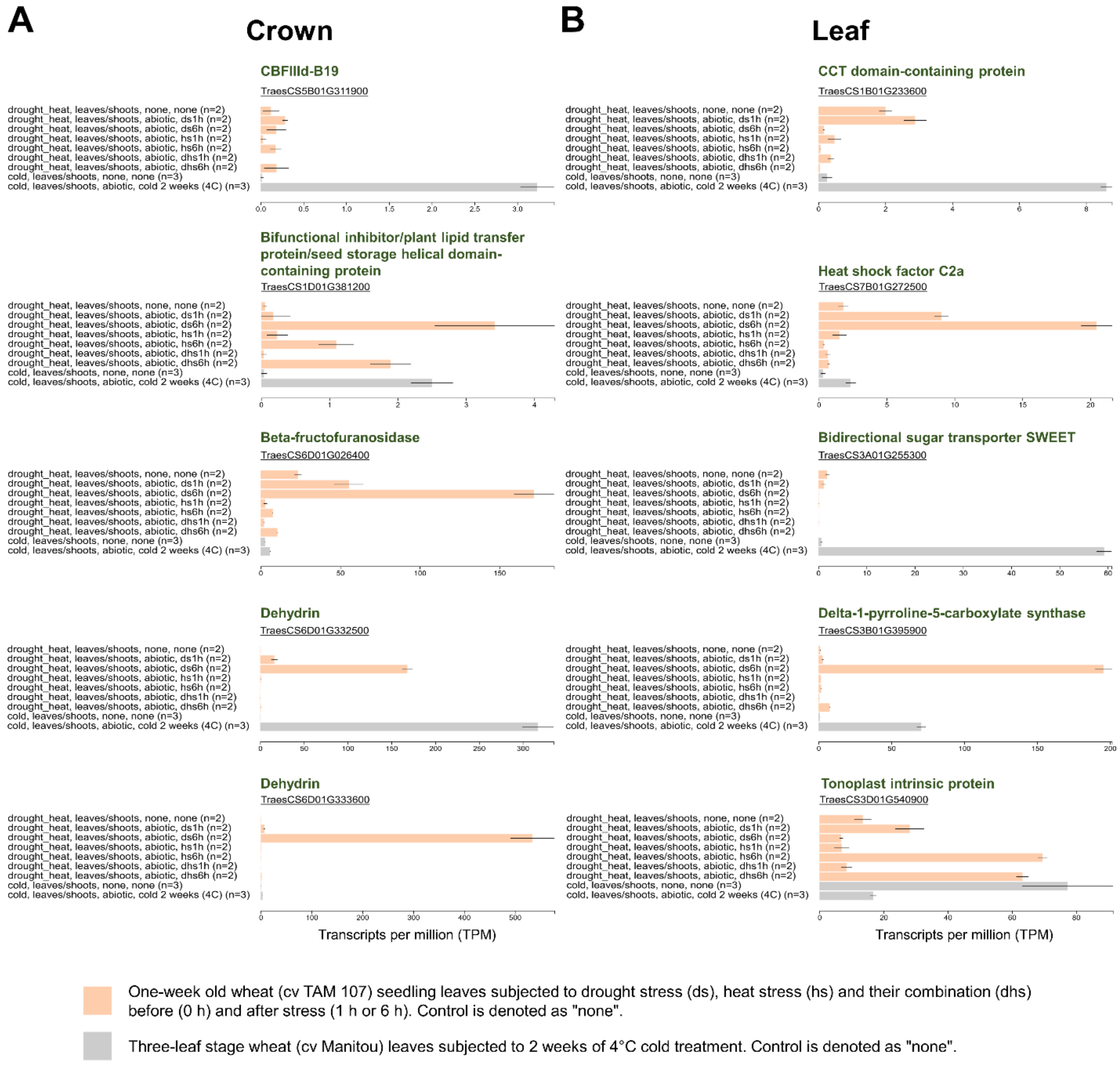

Supplementary Figure 2. The results of digital gene expression analysis performed on Wheat Expression Browser (Borrill et al., 2016), using the available RNA-seq data from wheat abiotic stress studies on drought, heat (Liu et al., 2015), and cold (Li et al., 2015). Five protein-encoding genes significantly expressed in crown (**A**) and leaf (**B**) tissues each were chosen. Provided are the RefSeq v1.0 gene IDs and corresponding UniProt KB descriptions. These genes are marked in **Figure 2** (heatmap) of the main manuscript.

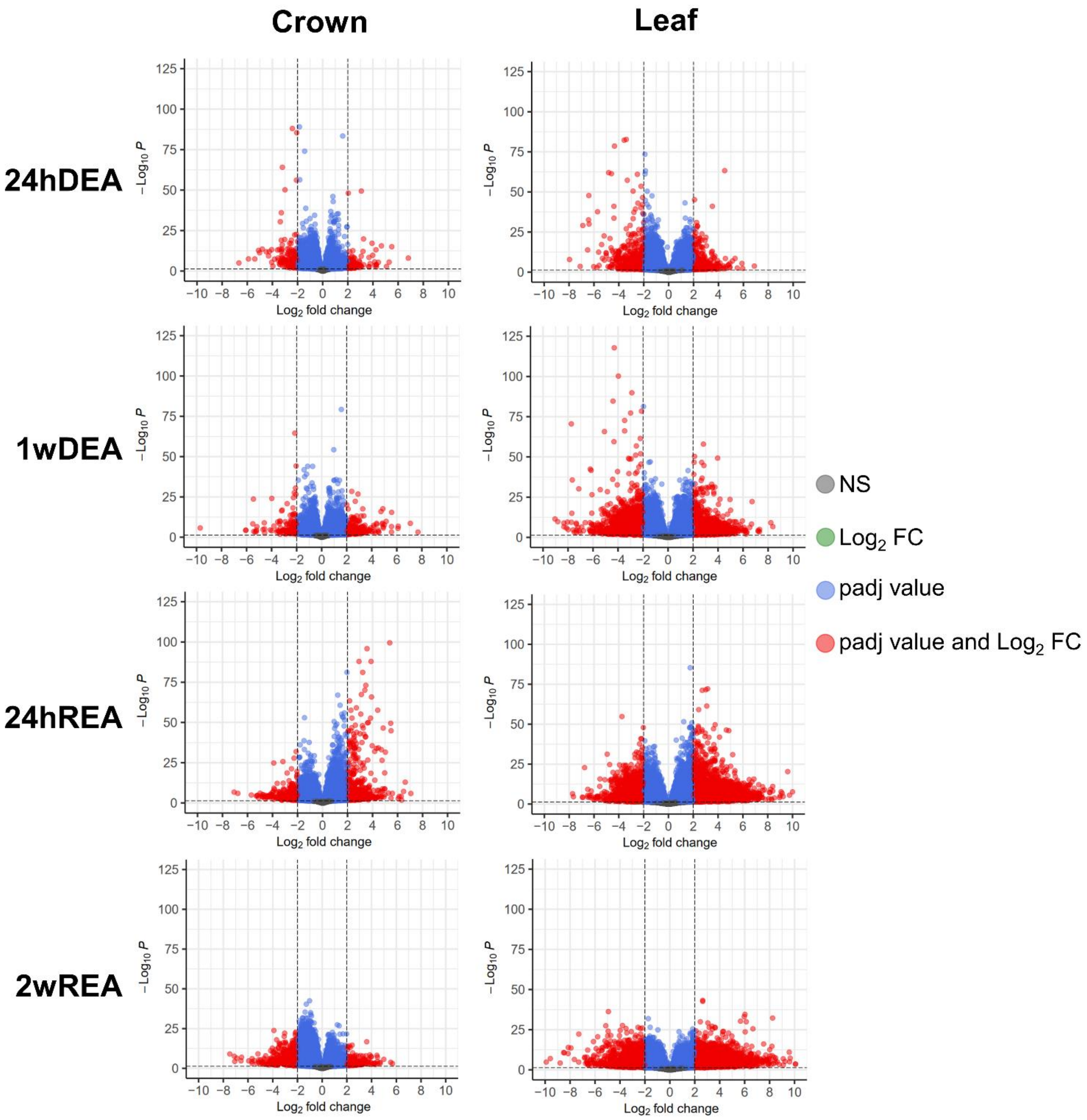

Supplementary Figure 3. Volcano plots, depicting the changes in gene expression during deacclimation (DEA) and reacclimation (REA) in crown and leaf tissues. All sampling points were compared to the 7 weeks of cold acclimation at 2 °C (7wCA) timepoint in each corresponding tissue. Fold change cutoff = ±2, adjusted p-value (padj) cutoff = 0.05. 24hDEA – 24 hours of deacclimation at 10 °C; 1wDEA – 1 week of deacclimation at 10 °C; 24hREA – 24 hours of reacclimation at 2 °C; 2wREA – 2 weeks of reacclimation at 2 °C.

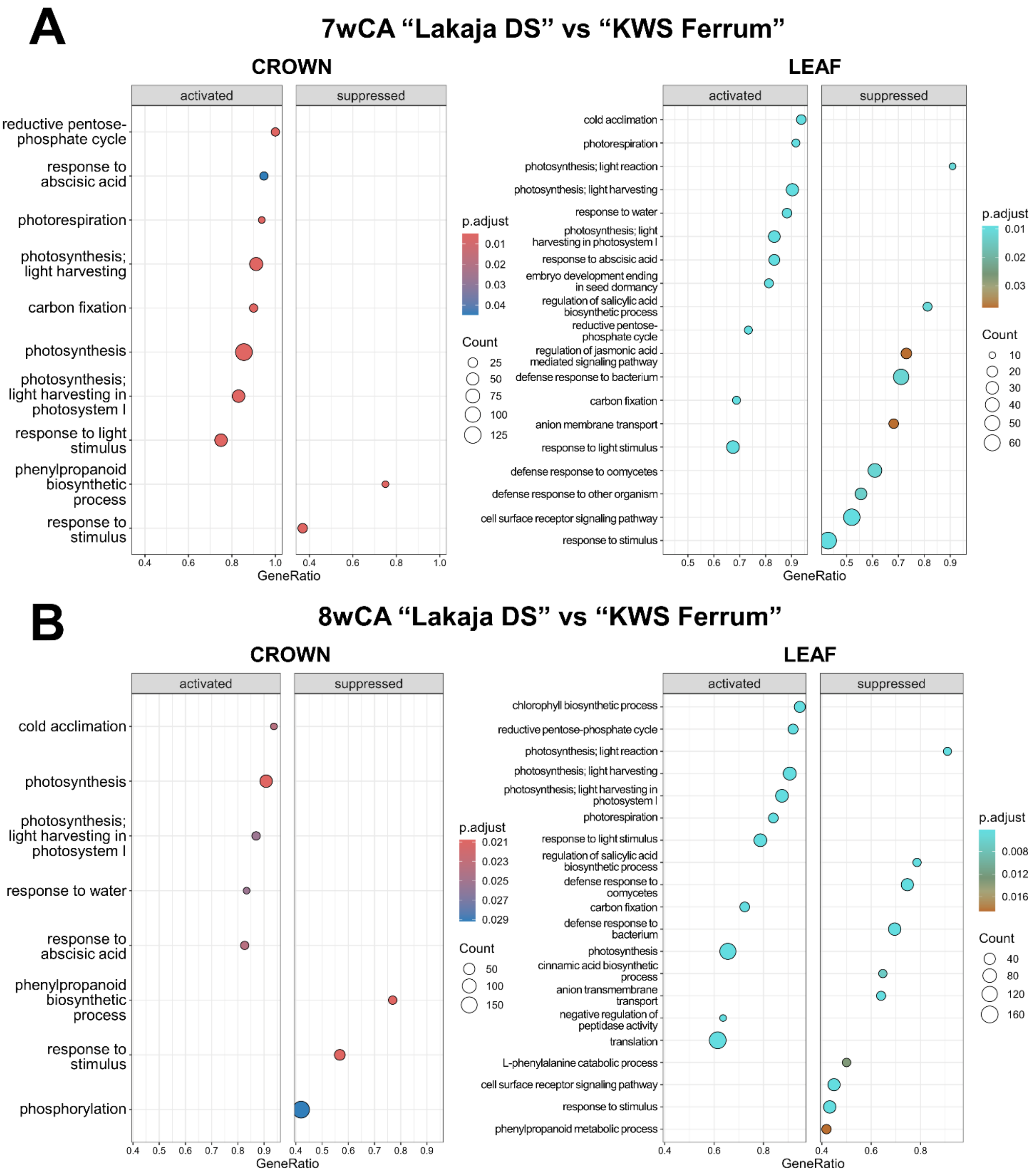

Supplementary Figure 4. Gene ontology (GO) analysis of gene expression data in crown and leaf tissue of “Lakaja DS” compared to “KWS Ferrum” after 7 weeks of cold acclimation (7wCA) (**A**) and 8 weeks of cold acclimation (8wCA) (**B**). BP (biological process) terms were used. p.adjust – adjusted p value, count – the number of genes in the expressed gene-set; GeneRatio – the ratio of number of genes in the expressed gene-set to the number of all genes in the full gene-set.

References

Borrill, P., Ramirez-Gonzalez, R., and Uauy, C. (2016). expVIP: a Customizable RNA-seq Data Analysis and Visualization Platform. *Plant Physiol.* 170, 2172–2186. doi:10.1104/pp.15.01667.

Li, Q., Zheng, Q., Shen, W., Cram, D., Fowler, D. B., Wei, Y., et al. (2015). Understanding the Biochemical Basis of Temperature-Induced Lipid Pathway Adjustments in Plants. *Plant Cell* 27, 86–103. doi:10.1105/tpc.114.134338.

Liu, Z., Xin, M., Qin, J., Peng, H., Ni, Z., Yao, Y., et al. (2015). Temporal transcriptome profiling reveals expression partitioning of homeologous genes contributing to heat and drought acclimation in wheat (*Triticum aestivum* L.). *BMC Plant Biol.* 15, 152. doi:10.1186/s12870-015-0511-8.
